# Augmented Cardiac Mitochondrial Capacity in High Capacity Aerobic Running “Disease-Resistant” Phenotype at Rest Is Lost Following Ischemia Reperfusion

**DOI:** 10.3389/fcvm.2021.752640

**Published:** 2021-11-03

**Authors:** Musaad B. Alsahly, Madaniah O. Zakari, Lauren G. Koch, Steven Britton, Laxmansa C. Katwa, Kelsey Fisher-Wellman, Robert M. Lust

**Affiliations:** ^1^Department of Physiology, Brody School of Medicine, East Carolina University, Greenville, NC, United States; ^2^East Carolina Diabetes and Obesity Center, East Carolina University, Greenville, NC, United States; ^3^Department of Physiology, College of Medicine, King Saud University, Riyadh, Saudi Arabia; ^4^Department of Physiology, College of Medicine, Taibah University, Medina, Saudi Arabia; ^5^Department of Physiology and Pharmacology, University of Toledo, Toledo, OH, United States; ^6^Departments of Anesthesiology and Molecular and Integrative Medicine, University of Michigan, Ann Arbor, MI, United States

**Keywords:** aerobic capacity, HCR, LCR, intrinsic, coronary occlusion, mitochondria, energetics

## Abstract

**Rationale:** Regular active exercise is considered therapeutic for cardiovascular disease, in part by increasing mitochondrial respiratory capacity, but a significant amount of exercise capacity is determined genetically. Animal models, demonstrating either high capacity aerobic running (HCR) or low capacity aerobic running (LCR) phenotypes, have been developed to study the intrinsic contribution, with HCR rats subsequently characterized as “disease resistant” and the LCRs as “disease prone.” Enhanced cardioprotection in HCRs has been variable and mutifactoral, but likely includes a metabolic component. These studies were conducted to determine the influence of intrinsic aerobic phenotype on cardiac mitochondrial function before and after ischemia and reperfusion.

**Methods:** A total of 34 HCR and LCR rats were obtained from the parent colony at the University of Toledo, housed under sedentary conditions, and fed normal chow. LCR and HCR animals were randomly assigned to either control or ischemia-reperfusion (IR). On each study day, one HCR/LCR pair was anesthetized, and hearts were rapidly excised. In IR animals, the hearts were immediately flushed with iced hyperkalemic, hyperosmotic, cardioplegia solution, and subjected to global hypothermic ischemic arrest (80 min). Following the arrest, the hearts underwent warm reperfusion (120 min) using a Langendorff perfusion system. Following reperfusion, the heart was weighed and the left ventricle (LV) was isolated. A midventricular ring was obtained to estimate infarction size [triphenyltetrazolium chloride (TTC)] and part of the remaining tissue (~150 mg) was transferred to a homogenation buffer on ice. Isolated mitochondria (MITO) samples were prepared and used to determine respiratory capacity under different metabolic conditions. In control animals, MITO were obtained and prepared similarly immediately following anesthesia and heart removal, but without IR.

**Results:** In the control rats, both resting and maximally stimulated respiratory rates were higher (32 and 40%, respectively; *p* < 0.05) in HCR mitochondria compared to LCR. After IR, resting MITO respiratory rates were decreased to about 10% of control in both strains, and the augmented capacity in HCRs was absent. Maximally stimulated rates also were decreased more than 50% from control and were no longer different between phenotypes. Ca^++^ retention capacity and infarct size were not significantly different between HCR and LCR (49.2 ± 5.6 vs. 53.7 ± 4.9%), nor was average coronary flow during reperfusion or arrhythmogenesis. There was a significant loss of mitochondria following IR, which was coupled with decreased function in the remaining mitochondria in both strains.

**Conclusion:** Cardiac mitochondrial capacity from HCR was significantly higher than LCR in the controls under each condition. After IR insult, the cardiac mitochondrial respiratory rates were similar between phenotypes, as was Ca^++^ retention capacity, infarct size, and arrhythmogenicity, despite the increased mitochondrial capacity in the HCRs before ischemia. Relatively, the loss of respiratory capacity was actually greater in HCR than LCR. These data could suggest limits in the extent to which the HCR phenotype might be “protective” against acute tissue stressors. The extent to which any of these deficits could be “rescued” by adding an active exercise component to the intrinsic phenotype is unknown.

## Introduction

Cardiovascular disease (CVD) remains the leading cause of morbidity and mortality in developed countries (1). The most common cause of cardiac injury is ischemic heart disease secondary to progressive coronary occlusion and is complicated frequently by complications such as ventricular arrhythmias and congestive heart failure (2). The most effective therapy, thus, far for limiting ischemic injury is early reperfusion, but reperfusion in itself has risks (3–5) to the extent that acute coronary occlusion and therapeutic intervention are collectively considered to produce an aggregate phenomenon recognized as ischemia-reperfusion (IR) injury (6–9).

Continuing research is aimed to explore the therapeutic interventions against IR injury. Although numerous pharmacological and preconditioning approaches to cardioprotection have been explored, regular exercise participation is recognized as an important, cost-effective, and safer lifestyle intervention in the prevention and treatment of IR injury (10–14). Redundant protective effects are evident in the exercised heart, namely, increased levels of heat shock proteins (15), altered nitric oxide (NO) signaling (16–18), enhanced Ca^2+^ handling proteins (19), improved ATP-sensitive potassium channels (20), and enhanced endogenous antioxidant (13, 21).

As an important site for ATP production *via* oxidative phosphorylation, mitochondria are critical in regulating normal cardiac metabolism and play a key role in the susceptibility of the heart to IR injury (22–28). The heart is an organ with high metabolic demand. Providing the myocardium with adequate coronary perfusion and oxygen delivery is crucial, but enhanced mitochondrial capacity also is essential. Mitochondrial respiratory rate and enzyme activities are the major elements that drive the oxidative phosphorylation process and structural integrity of the mitochondria. Myocardial IR can cause severe effects on mitochondrial homeostasis which dramatically affects mitochondrial function and survival (29–32). In addition to the detrimental effects of impaired mitochondrial energy production, mitochondrial ionic imbalance, and cell stress signaling can cause mitochondrial-mediated cell death (33–37).

Myocardial IR injury is an important cause of impaired heart function in the early postoperative period after cardiac surgery and acute myocardial ischemia. Growing evidence has become available supporting a crucial role of mitochondrial dysfunction in myocardial IR injury. Mitochondrial dysfunction during ischemia is a major mechanism that contributes to cardiomyocytes damage during IR (24, 26–31). Increased reactive oxygen species (ROS) generation, defects in electron transport chain activity and oxidative phosphorylation process, impaired respiratory chain complexes activity, opening of the mitochondrial permeability transition pore (mPTP), and release of cytochrome care considered contributing factors in mitochondrial dysfunction associated with heart IR (30–36).

Stunned (reversibly injured and nonfunctioning) myocardium displays relatively excess oxygen consumption for a specified rate of contractile work and, therefore, has a declined mechanical efficiency, which may be due to a rapid recovery of the intracellular pH during reperfusion (6). Once perfusion is restored, the intracellular increased accumulation of H^+^ during ischemia is transported into the extracellular space to normalize the pH in exchange for Na^+^ *via* Na^+^/H^+^ exchanger, while ATP depletion inactivates Na^+^/K^+^-ATPase. The combined effect results in an increase in intracellular Na^+^ which, in turn, activates the sarcolemmal 2Na^+^/Ca^2+^ exchanger, resulting in the exchange of intracellular Na^+^ with extracellular Ca^2+^. A high rate of 2Na^+^/Ca^2+^exchange can finally lead to Ca^2+^ overload which, in turn, induce arrhythmogenesis, myocardial stunning, contracture, and ultimately apoptotic or autophagic cell death. Fluctuations in Ca^2+^from the sarcoplasmic reticulum during reperfusion stimulate the opening of the mPTP (29, 30). Opening of the mPTP leads to rapid dissipation of the membrane potential gradient which is essential for the synthesis of ATP, water enters through the open pore causing mitochondrial swelling and lysis triggering apoptosis and cell death (28–30).

The proposed mechanisms underlying exercise-induced cardioprotection in IR are numerous. Some are systemic (12), some are vascular (12–14, 21), some are neural (38, 39), some are structural (9), and some are energetic/metabolic (40, 41) including expression of selected mitochondrial proteins resulting in a mitochondrial phenotype that is resistant to IR-induced injury (13, 19). While these studies generally support exercise-induced adaptations that produce resistance to injury, few address the mitochondrial functional consequences following an injury.

Although regular exercise training is recognized as an important lifestyle intervention in the prevention and treatment of CVD and IR injury, not all the individuals experience the same benefits from participating in the exercise. There is a paradox that some individuals with many modifiable risk factors (hypertension, diabetes, and obesity), and who do not exercise, also do not get CVD, while others who have no risk factors, and exercise regularly, and still experience adverse cardiac outcomes, suggesting a significant intrinsic component to the exercise effect. It has been estimated that up to 70% of the variation in exercise capacity is due to the intrinsic genetic component (42). Thus, studying the differential impacts of intrinsic aerobic exercise capacity after cardiac IR injury using intrinsic aerobic phenotype rats bred for low and high aerobic running capacity would provide a better platform for understanding the influences of intrinsic aerobic capacity on cardiac metabolic capacity and mitochondrial adaptive response pre- and post-IR injury in these phenotypes.

Koch and Britton used phenotypic selection based on treadmill running time at 11 weeks of age in an outbred rat strain (NIN:N) to create divergent strains that have become known as high capacity aerobic running (HCR) and low capacity aerobic running (LCR) rats (43–47). The HCR animals generally are characterized as “disease resistant,” while the LCR animals are characterized as disease prone. Interestingly, the HCR/LCR strains demonstrate many of the same traits related to CVD that had been previously associated with active exercise (48–52) including some mitochondrial/metabolic effects (53, 54). Still, cardioprotection in the HCRs is not always observed (55–57), and while LCR did associate with a higher incidence of pump failure, it did not associate with multiorgan system failure in hemorrhagic shock (58). We have previously reported that cardioprotection in HCRs was present but limited, was likely intrinsic to the tissue, and could be overwhelmed by IR severity (57). Given the consistent metabolic differences that characterized HCR and LCR, the current studies were designed to identify the relative influence of aerobic phenotype on the impact of IR on subsequent mitochondrial function.

## Materials and Methods

### Animal Strains

A total of 34 HCR and LCR female rats from generation 32 were obtained from the parent colony at the University of Toledo. The protocol for generating the animal model has been described in detail previously (43–46). Briefly, starting with an outbred founder population (N: NIH stock), two-way selective breeding using run time until exhaustion on a graded treadmill exercise test as selection criteria, was used to create low capacity runner (LCR) and high capacity runner (HCR) strains. A total of 13 animals of each sex with the shortest run times and 13 animals of each sex with the highest run times were used as the founding population of LCR and HCR cohorts. Well-managed breeding strategies within each cohort were used to create subsequent generations that were increasingly divergent in total running capacity. Upon arrival at this location, animals were maintained in quarantine under standard husbandry conditions. Animal procedures were conducted following the American Physiological Society guidelines for the humans and safe use of animals and all the protocols involving animals in these experiments were approved by the East Carolina University Animal Care and Use Committee.

### Cardiac Ischemic-Reperfusion Injury

Rats were anesthetized with an intraperitoneal injection of ketamine/xylazine (90/10 mg/kg ip, Patterson Veterinary Supply, Greeley, Colorado, USA). Once appropriate anesthetic depth was achieved, the thorax was opened and the heart was excised rapidly. The aortic root was cannulated and the coronary circulation was flushed immediately with warm saline. After the saline flush, the control hearts were flushed again with cold saline and transferred to buffer to being mitochondrial isolation. The IR hearts were immediately arrested with 10 ml of iced St Thomas' cardioplegic solution (NaCl 110.0 mM, NaHCO_3_ 10.0 mM, KCl 16.0 mM, MgCl_2_ 16.0 mM, CaCl_2_ 1.2 mM, and pH 7.8) at 4°C and the hearts were then stored in this solution at the same temperature for 80 min. After 80 min of cold global ischemic arrest, the aorta was cannulated and the heart was immediately perfused retrogradely on a Langendorff perfusion apparatus with Krebs–Henseleit buffer (KHB) for 120 min at 37°C (KHB composition (mM): NaCl 118; KCl 4.7; MgSO_4_ 1.2; KH_2_PO_4_ 1.2; NaHCO_3_ 25; CaCl_2_ 1.4; glucose 11; and pH 7.3–7.4). Perfusion was gravity-fed constant pressure maintained at 80 mm Hg, established by the appropriate height of the perfusion reservoir above the heart. Coronary flow and heart rhythm were monitored throughout the reperfusion period. The Langendorff system is widely used because of the ability to control a large number of performance variables (preload, rate, and afterload), but it also has the disadvantage of not actively pumping a volume. In addition, since the system is crystalloid perfused typically, any circulating agents like pro- or anti-inflammatory cytokines are not present, nor are hormones or autonomic nervous influences. In this case, that can be an asset, since any of those can be changed by exercise and would not be confounding aspects of the study.

### Tissue Isolation and Infarct Size Quantification

Following reperfusion, the IR hearts were taken off the cannula and weighed. The right ventricle and atrial tissue were removed and a midventricular ring was obtained from the left ventricle (LV) for infarct size quantification, while the balance of the LV was prepared for mitochondrial isolation, or for −80°C storage and future analysis. The midventricular ring was placed in a 0.1% triphenyltetrazolium chloride solution and incubated at 37°C for 10 min in a shaking water bath. Following incubation, both sides of the slice were photographed with a digital camera attached to a dissecting microscope. Images were quantified using Image J software where total area, lumen area, and infarcted area were measured and quantified as described previously (21, 30, 57).

### Mitochondria Isolation

Left ventricular mitochondria were isolated as described in Fisher-Wellman et al. (68). Briefly, one portion of isolated LV (~40 mg) was immediately placed in ice-cold buffer A [phosphate-buffered saline (pH = 7.4), supplemented with EDTA (10 mM)]. All the tissues were minced and resuspended in buffer C (MOPS (4-Morpholinepropanesulfonic acid, 50 mM; pH = 7.1), KCl (100 mM), EGTA (egtazic acid, 1 mM), and MgSO_4_ (5 mM) supplemented with bovine serum albumin (BSA; 2 g/l)) and then homogenized *via* a Teflon pestle and borosilicate glass vessel. Tissue homogenates were centrifuged at 500 × g for 10 min at 4°C. Supernatant from each tissue was then filtered through thin layers of gauze and subjected to additional centrifugation at 10,000 × g for 10 min at 4°C. Mitochondrial pellets were washed in 1.4 ml of buffer B [MOPS (50 mM; pH = 7.1), KCl (100 mM), EGTA (1 mM), and MgSO_4_ (5 mM)], transferred to microcentrifuge tubes, and centrifuged at 10,000 × g for 10 min at 4°C. Buffer B was aspirated from each tube and final mitochondrial pellets were suspended in 100–200 μl of buffer B. Protein content was determined *via* the Pierce BCA protein assay. Functional assays involving isolated mitochondria were carried out in the following buffers: buffer D-potassium-MES (105 mM; pH = 7.2), KCl (30 mM), KH_2_PO_4_ (10 mM), MgCl_2_ (5 mM), EGTA (1 mM), BSA (2.5 g/l); buffer E-HEPES (20 mM; pH = 8.0), KCl (100 mM), KH_2_PO_4_ (2.5 mM), MgCl_2_ (2.5 mM), and glycerol (1%).

### Mitochondrial Respiratory Control (JO_2_)

High-resolution O_2_ consumption measurements were conducted at 37°C in 2 ml of assay buffer by using the Oroboros Oxygraph-2K (Oroboros Instruments, Innsbruck, Austria), as previously described (67). Briefly, isolated mitochondria (0.025 mg/ml) were added to assay buffer, supplemented with creatine (Cr; 5 mM), phosphocreatine (PCr; 1 mM), and creatine kinase (CK; 20 U/ml), followed by the addition of respiratory substrates then ATP (5 mM). Respiratory control was assessed through the sequential additions of PCr to final concentrations of 6 mM, 12 mM, 15 mM, and 21 mM before additions of 5 μM fluoro-carbonyl cyanide phenylhydrazone (FCCP). Calculation of ATP free energy of hydrolysis (ΔG_*ATP*_) was determined for each PCR concentration as previously described (68) using an online tool (Bioenergetic Calculators (dmpio.github.io)).

### Citrate Synthase Activity

Citrate synthase activity was measured using a standard, commercially available kit (Sigma, St Louis, Missouri, USA), according to the instructions of the manufacturer. The assay generates a colorimetric signal in proportion to the rate of conversion between acetyl-CoA and oxaloacetic acid and is read on a spectrophotometer.

### Ca^2+^ Retention Capacity

Calcium retention protocols were modified from Sloan et al. (30) where 0.5 mg mitochondria were suspended in an assay buffer containing: 125 mM KCl, 5 mM HEPES, 2 mM KH_2_PO_4_, and 1 mM MgCl_2_ (25°C, pH = 7.3). The fluorescent Ca^2+^ indicator, calcium green 5 N salt, was utilized to track changes in extramitochondrial calcium levels. Extramitochondrial calcium fluorescence was measured using a fluorescence spectrophotometer (Photon Technology International, Birmingham, New Jersey, USA), with excitation and emission wavelengths set to 506/532 nm, respectively. Calcium-induced mPTP opening experiments were performed under state 2 respiration conditions (5 mM glutamate/5 mM malate). Mitochondrial PTP opening was induced by subjecting mitochondria to sequential 50 nmol CaCl_2_ pulses every 3 min, which causes a repeated decrease in the fluorescent signal as Ca^2+^ is taken up by the mitochondria. Induction of mPTP was denoted by a sharp increase in extramitochondrial Ca^2+^fluorescence, representing the release of the accumulated Ca^2+^ from the mitochondrial matrix. Calcium-retention capacity was quantified as the amount of calcium needed to induce PTP opening (nmol CaCl_2_/mg mitochondria).

### Statistical Analysis

Statistical analysis was performed by using commercial software (GraphPad Prism, San Diego, California, USA). Data are expressed as means ± SD and a *p* < 0.05 was considered as statistically significant.

## Results

### Myocardial Infarct Size

**Figure 1:**
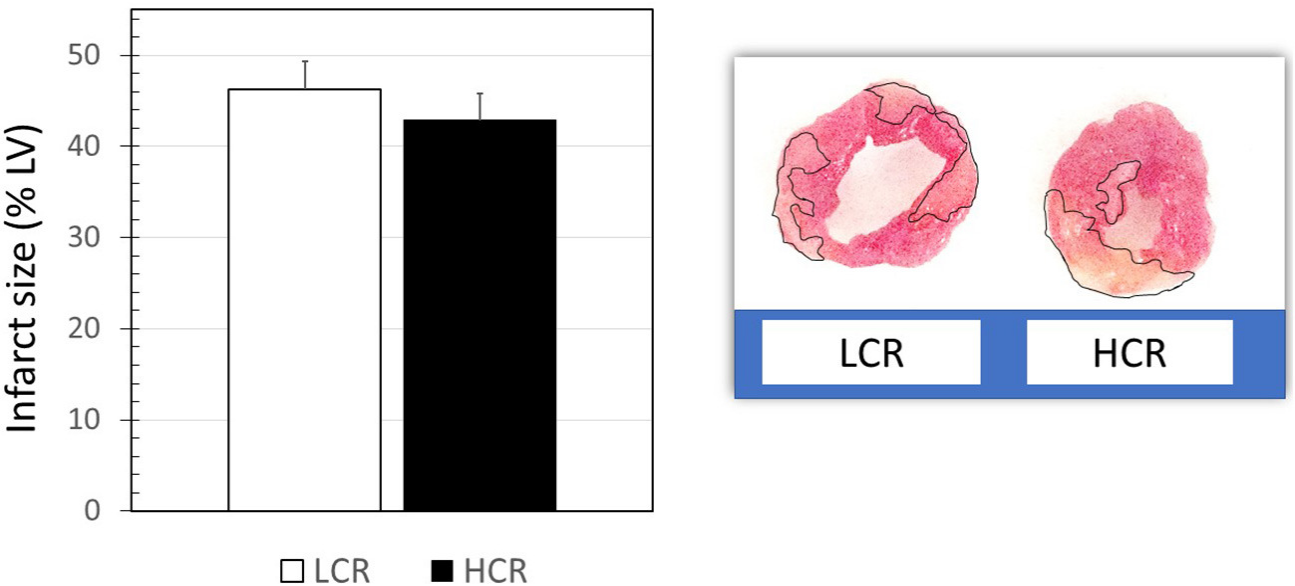
Examples of triphenyltetrazolium chloride (TTC)-stained myocardial sections, and graphical summary of group data for infarct size results (*n* = 9 per group).

Infarctions were somewhat larger than expected despite the relatively long ischemic times, considering the use of the cold cardioplegic solution. About 80 min of cold global ischemic arrest and 120 min of warm reperfusion, myocardial infarct size was not significantly different between LCRs and HCRs (47.2 ± 4.8 vs. 42.9 ± 5.2, respectively). Hearts from HCR and LCR animals showed different patterns of infarction, with HCR being more contiguous and LCR more diffuse, but relatively similar in the overall amount of tissue involved (Figure 1).

### Coronary Artery Flow

**Figure 2:**
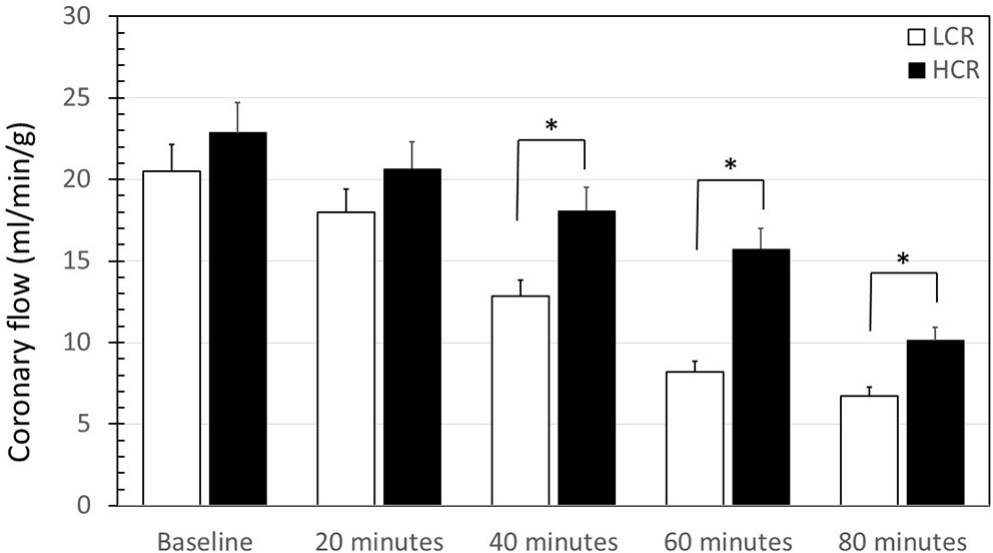
Summary of coronary flow results during reperfusion of low capacity aerobic running (LCR) and high capacity aerobic running (HCR) hearts. Data are expressed as mean ± SD. *n* = 9 per group. ^*^p < 0.05 in designated comparison.

When normalized to heart weight, baseline coronary flow at the onset of reperfusion was not different between LCR and HCR hearts (20.5 ± 3.2 vs. 23.6 ± 2.8, respectively). However, coronary flow decreased by 68% in LCRs and by 56% in HCRs over the 2 h of reperfusion and coronary flow decreased more quickly in the LCRs compared to the HCRs, becoming significantly different at 40 min of reperfusion (Figure 2). In a constant pressure system, the decreasing flow is consistent with an increased coronary vascular resistance. Still, there was a substantial drop in coronary flow in both groups, and the relatively higher/less diminished flow, was not enough to produce any difference in overall infarction size.

### Reperfusion Arrhythmia

**Figure 3:**
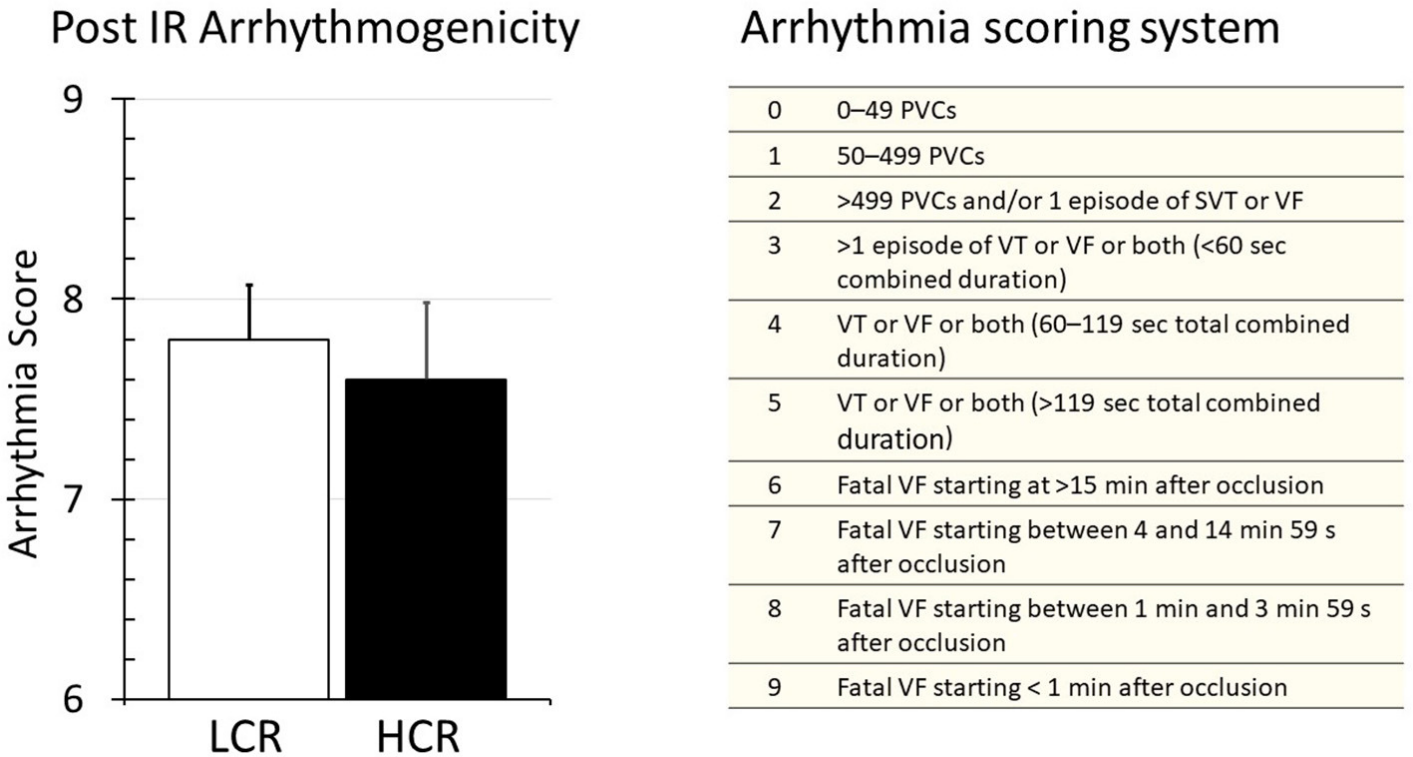
Summary of arrhythmia results during reperfusion of LCR and HCR hearts (left panel), and the arrhythmia scoring system that was used (right panel) to generate the data. Data are expressed as mean ± SD. *n* = 9 per group. ^*^p < 0.05 in designated comparison.

Upon harvest, the hearts were placed immediately into the cold storage solution, so there were no baseline rhythm measurements. The first perfusion on the column constituted the onset of rewarming and reperfusion. Arrhythmia was evaluated followed by the guidelines established by the Lambeth Conventions and graded by using a numerical scoring system as described previously (30). Fibrillation was a uniform finding early in reperfusion with similar frequency and severity in both LCR and HCR hearts (Figure 3).

### Mitochondrial Respiratory Capacity

#### Control Comparisons

**Figure 4:**
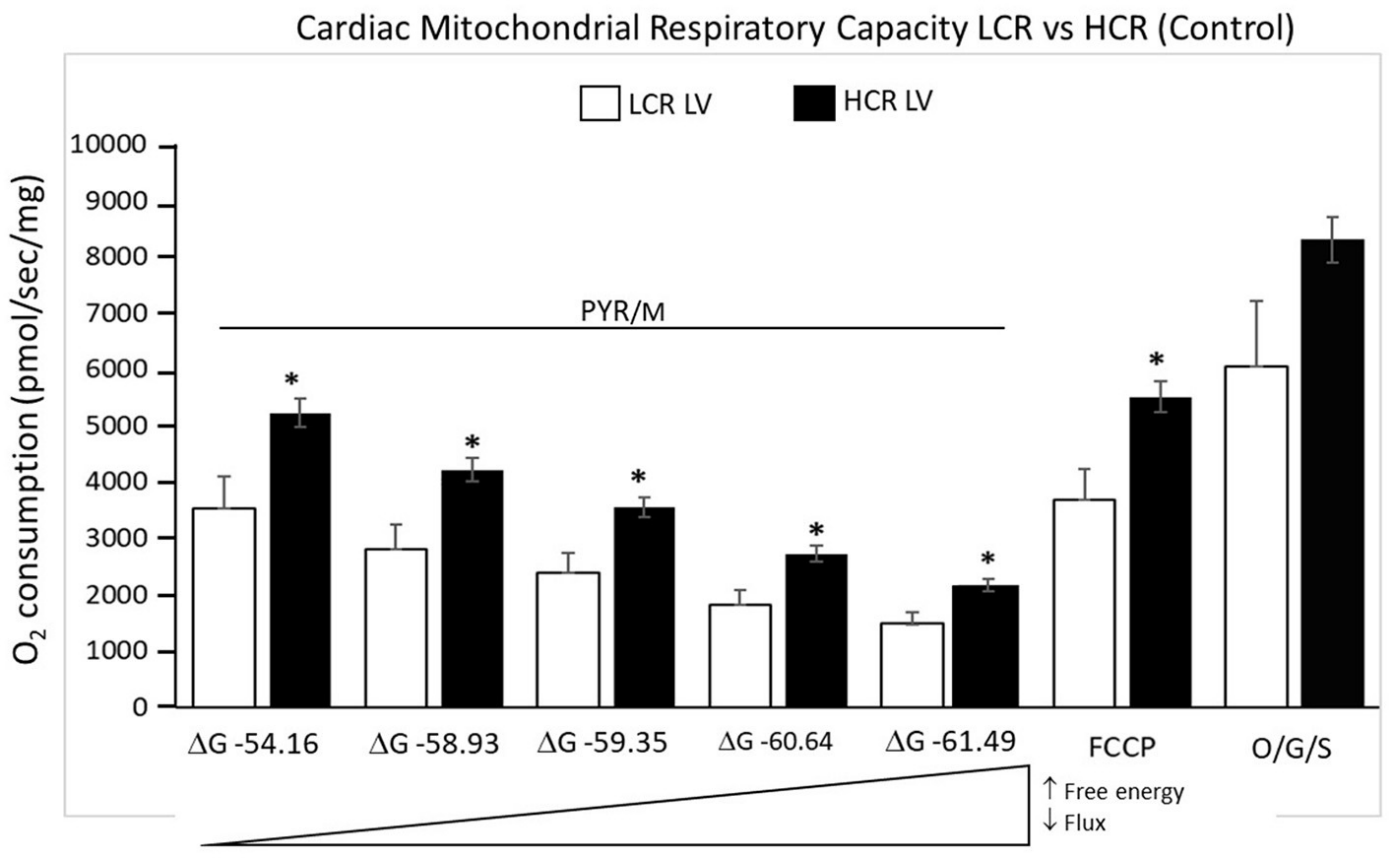
Summary of mitochondrial respiration results in samples isolated from HCR and LCR control ventricles. The top inset shows representative tracing, while the graphic summarizes group results. Data are expressed as mean ± SD. *n* = 8 per group. ^*^p < 0.05 in designated comparison.

Cardiac mitochondria respiratory rates were 32% higher at rest in HCR and more than 40% higher under maximally stimulated conditions, compared to LCR (both *p* < 0.05). Generally, HCR mitochondria showed significantly higher mitochondria respiration with all substrates compared to LCR. The addition of the uncoupling agent, carbonyl cyanide 4-(trifluoromethoxy) phenylhydrazone (FCCP) (1 μM), at the end of the respirometry experiments rescued absolute rates of oxygen consumption (Figure 4).

#### Post-ir Injury Comparisons

**Figure 5:**
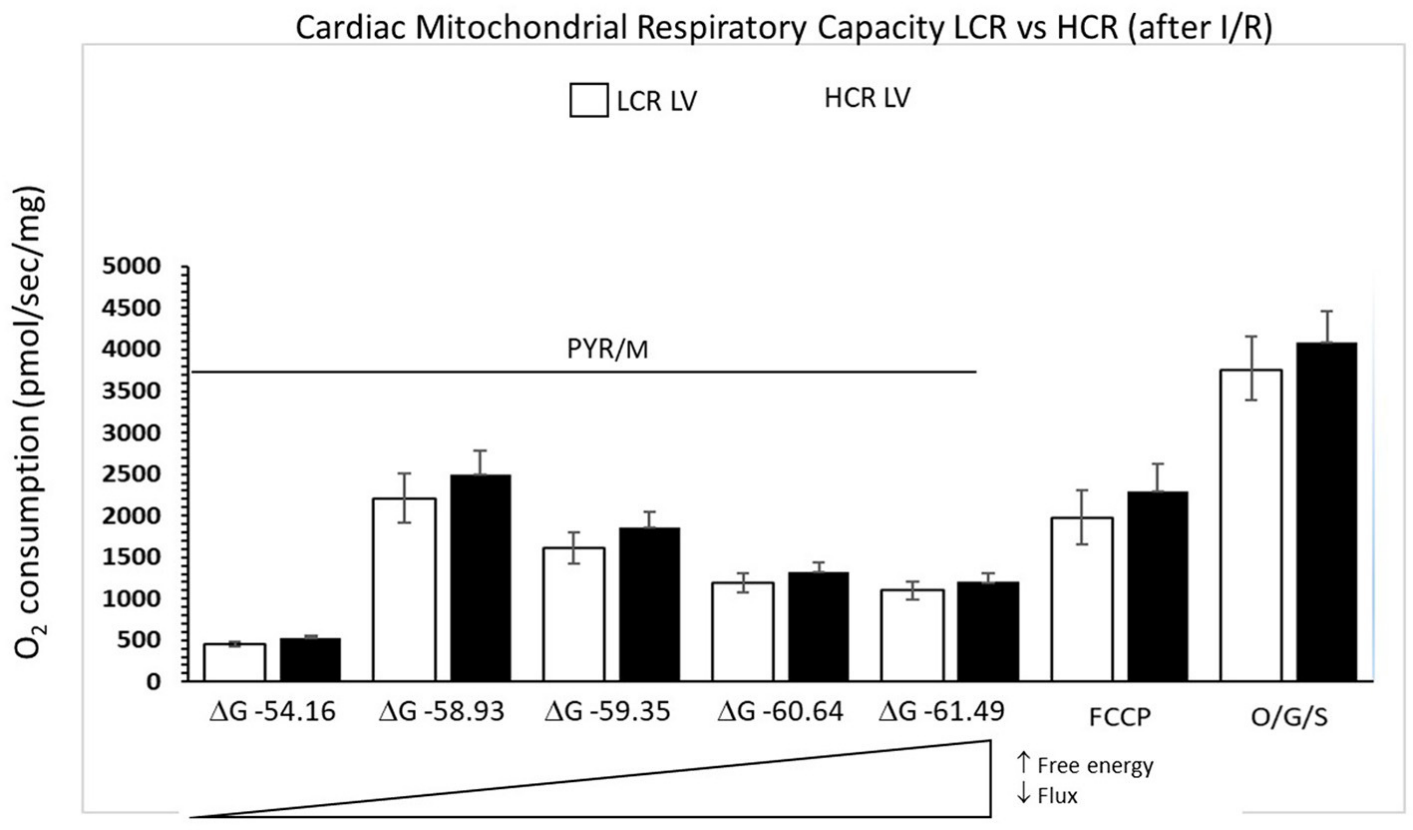
Summary of mitochondrial respiration results in samples isolated from HCR and LCR ventricles following IR. The top inset shows representative tracing, while the graphic summarizes group results. Data are expressed as mean ± SD. *n* = 9 per group. ^*^p < 0.05 in designated comparison.

**Figure 6:**
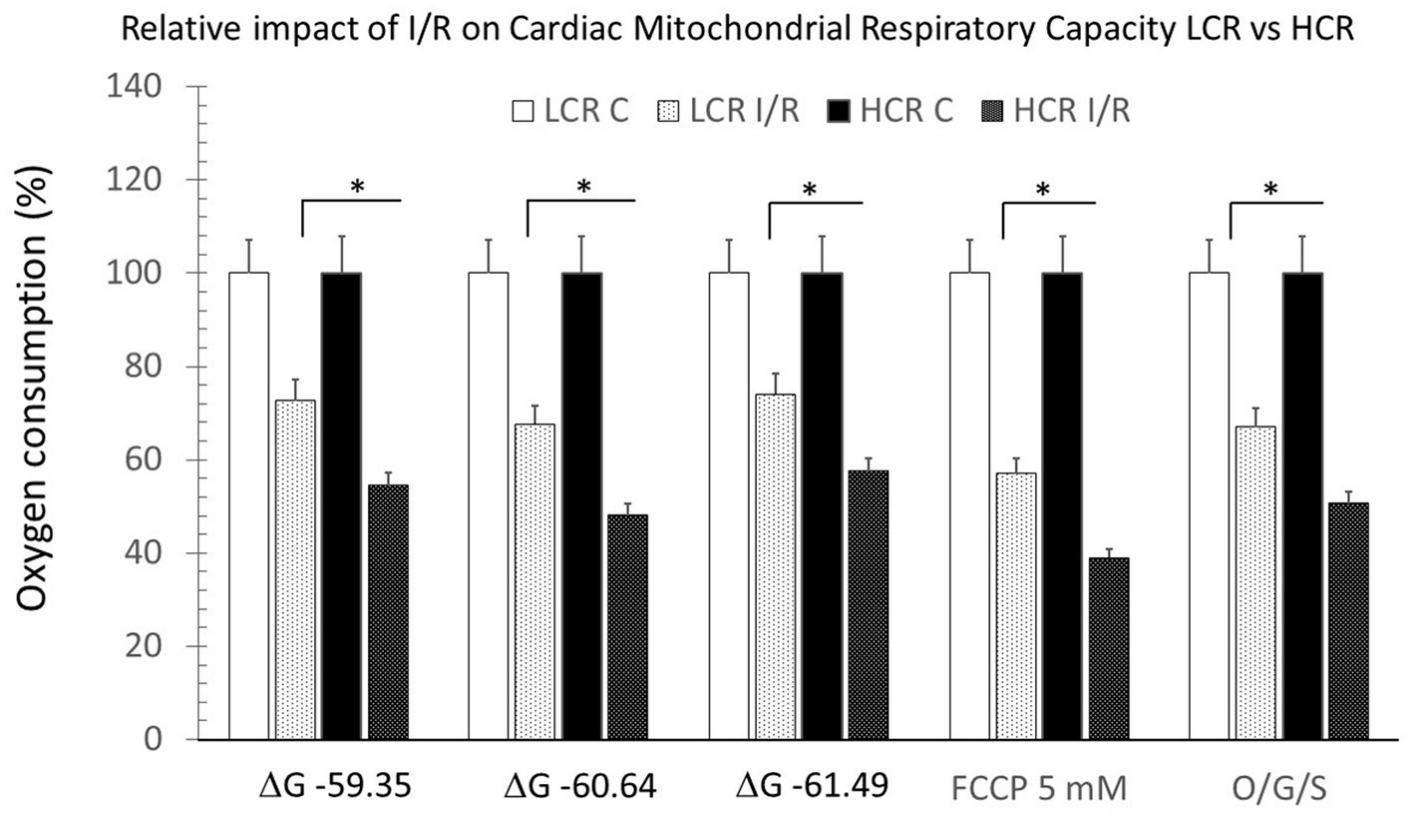
Summary of mitochondrial respiration, comparing results within and between phenotypes, under control and post-ischemia-reperfusion (IR) conditions. Data are expressed as mean ± SD. *n* = 8 per group for controls and 9 per group for IR. ^*^p < 0.05 in designated comparison.

In contrast to control experiments, HCR mitochondria no longer showed relatively increased respiratory capacity compared to LCR (Figure 5). Comparing control to post-I/R under pyruvate/malate conditions, respiratory rates were reduced in both phenotypes to levels <10% of baseline (Figure 6). There was recruitable respiratory capacity in both HCR and LCR postischemic mitochondria, but the differences between phenotypes that been observed across the spectrum of substrate conditions were no longer present after ischemia. In fact, HCRs showed a greater loss of respiratory capacity in response to FCCP than the LCR (Figure 6).

**Figure 7:**
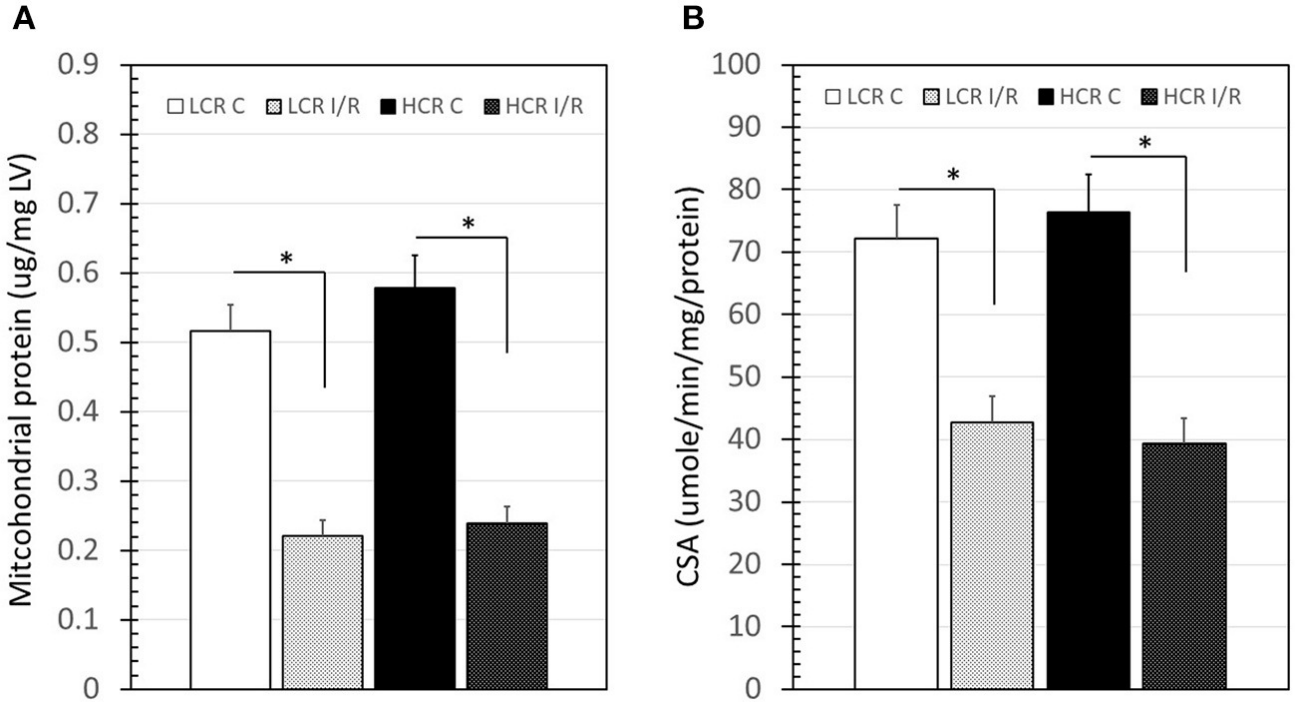
Summary of mitochondrial protein “yield” from left ventricle (LV) samples (left panel) and citrate synthase activity (CSA, right panel) before and after IR. Data are expressed as mean ± SD. *n* = 8 per group for controls and 9 per group for IR. ^*^p < 0.05 in designated comparison.

The amount of mitochondrial protein harvested from LV tissue samples (w/w) was not different between HCR and LCR under control conditions (Figure 7A). Following cold storage ischemia and rewarming/reperfusion, there was a significant decrease in mitochondrial protein in both strains (*p* < 0.05), consistent with a loss of mitochondria. However, the decrease was similar in both groups (Figure 7A). Interestingly, the citrate synthase activity of the remaining mitochondria following reperfusion also was reduced comparably in both groups, consistent with possible functional impairment in surviving mitochondria (Figure 7B).

#### Ca^2+^ Retention Capacity

**Figure 8:**
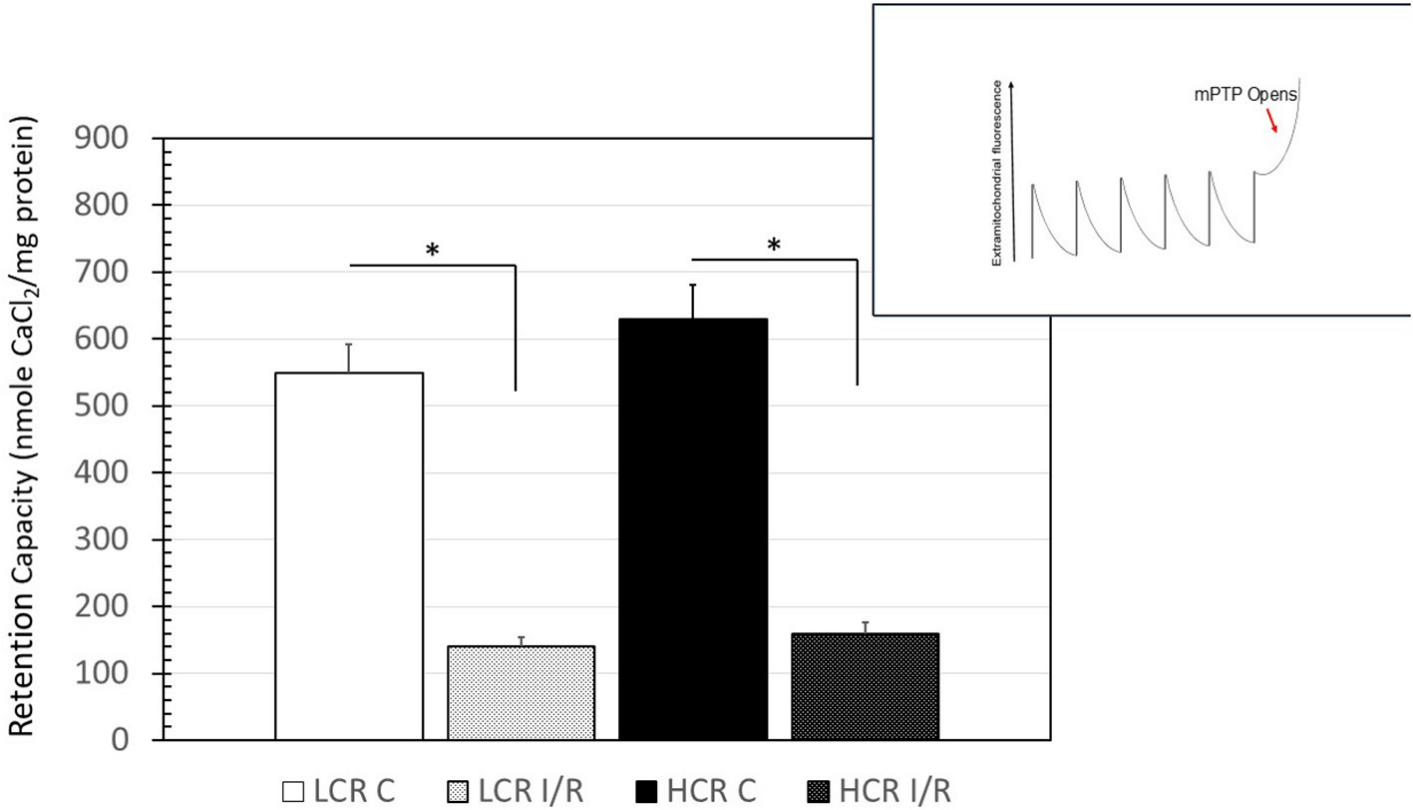
Summary of results for the calcium-retention experiments. Inset is a representation of the tracings obtained, while the chart is a graphical summary of the group data. Arrow in inset indicates the change in calcium uptake that occurs when the transition pore opens. Data are expressed as mean ± SD. *n* = 8 per group for controls and 9 per group for IR. ^*^p < 0.05 in designated comparison.

There were no differences in baseline mitochondrial calcium retention. Similar to the respiratory deficits that occurred in mitochondria after I/R, the ability to retain calcium was also reduced significantly following ischemia. On average, the permeability transition pore (PTP) opened with about 75% less calcium than it did before the I/R insult but the impairments were similar in both the phenotypes (Figure 8).

## Discussion

The results of this study showed that while there was increased mitochondrial capacity in HCRs compared to LCRs, the increased cellular respiratory capacity did not translate to reduced injury from ischemic arrest and reperfusion even with the benefit of supplemental cardioprotection by using established cardioplegia regimens. Moreover, there was accelerated relative loss of mitochondrial respiratory capacity in the HCRs compared to the LCRs, even though coronary blood flow was better preserved during reperfusion in the HCRs compared to the LCRs. The lack of benefit despite better flow was a bit unexpected, as dynamic exercise has generally associated cardioprotection with improved perfusion. A decrease in infarct size after training was seen in rats subjected to a swim training regimen after permanent occlusion which the authors attributed to increased myocardial vascularity (59). In a much-cited paper, Brown et al. demonstrated that prolonged endurance training confers a cardioprotective effect against infarction in myocardium subjected to severe ischemia and subsequent reperfusion (21). In addition, they observed that during severe ischemia, coronary flow to regions of the myocardium outside the ischemic area at risk was better maintained in hearts isolated from endurance-trained rats. Furthermore, on reperfusion of the area at risk, the increase in flow to the previously ischemic region of the heart was markedly higher in hearts isolated from trained rats (21).

We have demonstrated that the cardioprotection associated with the HCR phenotype may be limited (57). We did not have sufficient animals available to complete a preliminary dose response study to determine the optimal cold storage time, and it is possible that despite the cardioplegia, the ischemic time was simply too long and overwhelmed and benefit the HCR phenotype might have conferred. However, the results are consistent with those reported by others working with the model (55, 56). Still, there are important differences worth noting. In particular, active exercise training programs produce improvements to ischemic tolerance *via* a host of mechanisms (5, 6, 9, 10, 14, 15), notably including increased mitochondrial functional capacity (19, 24, 59). Gallego-Selles et al. described experiments in humans subjects indicating that, at least in skeletal muscle, nrf2 is activated rapidly by exercise to exhaustion and can dynamically modulate ROS and mitochondrial-sourced proteins such as catalase (61). Flockhart demonstrated, also in human subjects, that participating in an intense, active exercise program produced functional impairment in the mitochondria that also was associated with at least a transient decrease in glucose tolerance (62). In our hands, despite an increased resting mitochondrial capacity in the HCRs, there was no protection against the ischemic insult. Together, it seems clear that there can be substantial differences in the role of mitochondria in the response to ischemia that can be influenced by the nature and duration of the exercise training and confounded by differences dictated by the intrinsic capacity for exercise, independent of actual active exercise. The importance and role of pathways such as nrf2/keap1/catalase and others in bridging the components of an exercise effect (intrinsic vs. active) remain to be determined.

Several previous experimental studies of myocardial ischemia generally have indicated a good correlation between disruption of complex I through a variety of mechanisms (16–18, 33, 41), as well as opening of the permeability transition pore, with outcomes in cardiac ischemia and reperfusion (23, 26, 28–30). Some have suggested that complex I is considered the main site of damage to the respiratory chain in IR, while downstream electron transport chains are relatively resistant to IR injury (60, 63, 64). Furthermore, Veitch and his colleagues (63) found a major decline in complex I activity in perfused rat hearts subjected to 20 min of global ischemia, and Cairns and his group (64) demonstrated that this damage was exacerbated by reperfusion.

In contrast to these studies, our results suggest a more generalized loss of mitochondrial respiratory capacity following IR. Perhaps that should not be surprising. Animals that have been selectively inbred using aerobic running capacity as the selection criteria create a strain optimized for performance when aerobic conditions exist. Ischemia certainly is not an aerobic condition. While it is possible that the HCR phenotype augments several factors that could delay the onset of ischemia and which would be protective (57), they may be maladapted if the transition to anaerobic conditions still occurs. Structural adaptations like increased capillary density or increased vasodilator capacity, which might have been beneficial in delaying the onset of ischemia, would also be factors that could aggravate the potential for reperfusion injury once perfusion was reestablished after an acute ischemic episode. When ample oxygen is present, cardiac mitochondria have greater substrate flexibility than many other tissues, meaning that cardiac mitochondria can respire effectively using a wide variety of substrates (67). Tolerance for IR may not be related so much to the capacity to respire using a given substrate, but more to the ability to switch from one substrate to another. With the newest approaches that have been developed (68), and the energy hypothesis that is being advanced by the HCR LCR work (46, 53), the HCR LCR phenotypes could provide interesting and valuable perspective on factors contributing to substrate preference. To the extent that HCR complex I respiratory capacity was impacted strongly by IR, and that there was a similarity between PTP function and infraction size in HCR and LCR, suggest that a more complex assessment of specific mitochondria adaptations in these strains might be warranted.

Mitochondria comprise a large fraction of the heart mass and are critical for the normal mechanical and electrophysiological function of the cardiomyocyte, playing roles that extend beyond bioenergetics and metabolism. Proper function is required to meet the high energetic demand of the cardiomyocyte and playing an essential role in controlling oxidative stress and Ca^2+^ handling (34). Ischemia-reperfusion injury increases the production of ROS and induces calcium overload into mitochondria (35) which can interact together to induce opening of the mPTP and, therefore, triggering apoptosis by promoting the release of proapoptotic proteins (i.e., cytochromec) and subsequent activation of the programmed cell (65, 66).

Our results indicate that intrinsic aerobic capacity must be tied to mitochondrial performance at a subcellular level. It also is clear that the dynamic role of mitochondria in cardiac ischemia, perfusion, and heart failure is only now beginning to be appreciated, but remains confused, perhaps in part because the underlying intrinsic elements have not been adequately considered in the assessment of the active exercise response. Models such as these HCR and LCR phenotypes should provide a unique window on how that background influences the extent to which inducible adaptive responses can be accomplished best using an active exercise regimen.

## Data Availability Statement

The raw data supporting the conclusions of this article will be made available by the authors, without undue reservation.

## Ethics Statement

The animal study was reviewed and approved by Institutional Animal Care and Use Committee (IACUC) East Carolina University.

## Author Contributions

RL participated in designing, conducting, analyzing, and writing. MA and MZ participated in conducting, analyzing, and writing. KF-W participated in designing, conducting, and analyzing. LCK participated in conducting and analyzing. LGK and SB provided animals and insight on study design. All authors contributed to the article and approved the submitted version.

## Conflict of Interest

The authors declare that the research was conducted in the absence of any commercial or financial relationships that could be construed as a potential conflict of interest.

## Publisher's Note

All claims expressed in this article are solely those of the authors and do not necessarily represent those of their affiliated organizations, or those of the publisher, the editors and the reviewers. Any product that may be evaluated in this article, or claim that may be made by its manufacturer, is not guaranteed or endorsed by the publisher.
