# Correction: Augmented cardiac mitochondrial capacity in high capacity aerobic running “disease-resistant” phenotype at rest is lost following ischemia reperfusion

**DOI:** 10.3389/fcvm.2026.1854980

**Published:** 2026-05-26

**Authors:** Musaad B. Alsahly, Madaniah O. Zakari, Lauren G. Koch, Steven Britton, Laxmansa C. Katwa, Kelsey Fisher-Wellman, Robert M. Lust

**Affiliations:** 1 Department of Physiology, Brody School of Medicine, East Carolina University, Greenville, NC, United States; 2 East Carolina Diabetes and Obesity Center, East Carolina University, Greenville, NC, United States; 3 Department of Physiology, College of Medicine, Taibah University, Medina, Saudi Arabia; 4 Department of Physiology and Pharmacology, University of Toledo, Toledo, OH, United States; 5 Departments of Anesthesiology and Molecular and Integrative Medicine, University of Michigan, Ann Arbor, MI, United States

**Keywords:** aerobic capacity, coronary occlusion, energetics, HCR, intrinsic, LCR, mitochondria

Affiliation Department of Physiology, College of Medicine, Taibah University, Medina, Saudi Arabia was omitted for author Madaniah O. Zakari. This affiliation has now been added for author Madaniah O. Zakari.

Author Madaniah O. Zakari was erroneously assigned to affiliation Department of Physiology, College of Medicine, King Saud University, Riyadh, Saudi Arabia. This affiliation has now been removed for author Madaniah O. Zakari.

The original version of this article has been updated.

